# Evaluation of posterior segment changes in pediatric asthma patients with and without inhaled corticosteroid therapy

**DOI:** 10.1038/s41598-025-14024-w

**Published:** 2025-08-06

**Authors:** Ulviye Kıvrak, Fatih Çiçek, Mehmet Tolga Köle, Büşra Kaya Adaş, İbrahim Kandemir

**Affiliations:** 1 Department of Ophthalmology, Kartal Dr Lütfi Kırdar City Hospital University of Health Sciences, Cevizli, D-100 Güney Yanyol, Cevizli Mevkii No:47, 34865 Kartal, Istanbul, Turkey; 2 Advanced Neurologıcal Sciences, Istanbul University Institute of Graduate Studies in Health Sciences, Istanbul, Turkey; 3 Department of Pediatrics, Istinye University, Istanbul, Turkey; 4 Department of Pediatrics, Ankara Etlik City Hospital, Ankara, Turkey; 5 Department of Pediatrics, Istanbul Health and Technology University, Faculty of Medicine, Istanbul, Turkey

**Keywords:** Asthma, Inhaled corticosteroid, Optic coherence tomography angiography, Vascular density, Retinal diseases, Vision disorders

## Abstract

This study aimed to evaluate and compare changes in the posterior segment of pediatric asthma patients, potentially associated with asthma or inhaled corticosteroids. A retrospective analysis was conducted on children aged 7–17 diagnosed with atopic asthma. The participants were categorized into groups: Group 1 (no inhaled corticosteroids) and Group 2 (inhaled corticosteroid treatment). A control group of healthy children was also included. Demographic data, clinical findings, and laboratory results (e.g., eosinophil count, IgE, CRP levels) were collected. Optical coherence tomography (OCT) and OCT angiography (OCTA) were used to measure posterior segment parameters. Asthma patients demonstrated a statistically significant reduction in subfoveal choroidal thickness, the superficial capillary plexus in the superior and nasal quadrants, the choriocapillaris in the foveal quadrant, and the peripapillary vascular density in the inferior and superior quadrants compared to controls. Inflammation markers such as eosinophil count and CRP showed significant correlations with changes in vascular density. Asthma, as a chronic inflammatory and hypoxic condition, can significantly affect posterior segment parameters, leading to potential visual function impairments in children. Regular monitoring with OCT and OCTA can help detect early microvascular changes, allowing for timely interventions to preserve visual health.

## Introduction

Asthma is a heterogeneous disease characterized by chronic airway inflammation, which manifests through respiratory symptoms such as wheezing, shortness of breath, chest tightness, and coughing^1^. Its prevalence in children varies across populations, ranging from 3 to 23.2%^2^. The pathophysiology of asthma involves the activation of type 2 CD4+ T-helper cells, as well as the release of cytokines, eosinophils, mast cells, macrophages, and epithelial cells, all of which contribute to the inflammatory process. Additionally, genetic predisposition, epigenetic mechanisms, and environmental factors play significant roles in the development and exacerbation of the disease^3,4^.

Asthma is not limited to the pulmonary tissues and airways; it has also been associated with other conditions such as gastroesophageal reflux, allergic rhinitis, obesity, depression, diabetes mellitus, atherosclerosis, and cardiovascular diseases. This comorbidity is thought to be related to the inflammatory processes and hypoxia that occur in asthma^5^. Asthma treatment follows an algorithmic approach tailored to the severity of the disease^6^. Inhaled corticosteroids are one of the most commonly used pharmacological agents in asthma management, as they reduce symptoms and exacerbations, improving quality of life^7^.

In asthma patients, an increase in myopia progression, reduction in contrast sensitivity, development of cataracts, glaucoma, visual field loss, central serous chorioretinopathy, and an increased incidence of age-related macular degeneration have been reported^8–12^. Some of these findings have been attributed to systemic inflammation, hypoxia, and deficiencies in vitamin A and copper observed in asthma patients, while others are linked to the inhaled corticosteroids used in asthma treatment^10–12^.

Optical coherence tomography (OCT) and optical coherence tomography angiography (OCTA) are practical, rapid, and non-invasive imaging techniques that enable the assessment of neuronal and vascular structures in the posterior segment^13^. The aim of this study was to evaluate and compare changes in posterior segment parameters in pediatric patients with asthma, potentially associated with the disease itself or the use of inhaled corticosteroid treatment, with healthy children as controls.

## Materials and methods

This retrospective study was conducted in accordance with the principles of the Helsinki Declaration by the Ophthalmology and Pediatric Allergy Clinics, with approval from the local ethics committee of our hospital (Dr. Lütfi Kırdar Kartal City Hospital ethical review committee, Protocol Number: 2024/010.99/3/22). The study included children diagnosed with asthma, followed up at the Pediatric Allergy Clinic between January 2023 and January 2024, as well as age- and gender-matched healthy controls. Due to the retrospective nature of the study, informed consent was not obtained.

The study included patients aged 7–17 years who were diagnosed with atopic asthma and had either at least one positive aeroallergen on skin prick testing or detectable levels of specific immunoglobulin E (IgE). Asthma was diagnosed in accordance with the GINA 2024 guidelines, based on the presence of respiratory symptoms such as episodic and variable shortness of breath, wheezing, cough, and chest tightness accompanied by variable expiratory airflow limitation, demonstrated by a significant bronchodilator or inhaled corticosteroid response, defined as an increase in forced expiratory volume in 1 s (FEV₁) by ≥ 12% or peak expiratory flow (PEF) by ≥ 20% as measured by spirometry^1^^,^^14^. Patients were divided into two groups. Group 1 consisted of patients with atopic asthma who were not receiving inhaled steroid treatment; Group 2 included patients who had been receiving inhaled fluticasone propionate (Flixotide, GlaxoSmithKline, Middlesex, UK) 250 μg daily for more than 1 year. In Group 2, to ensure the appropriate therapeutic dose reached the lungs, fluticasone propionate was administered using an aerochamber for all participants. The control group consisted of patients from the Ophthalmology Clinic who presented with minor symptoms, such as refractive errors, and had no history of respiratory diseases or parasitic infections. Patients with a refractive error of ≥ ± 3.00 diopters, a history of chronic diseases or genetic syndromes (other than atopic asthma), amblyopia, corneal pathology, retinal diseases, glaucoma, optic disc anomalies, uveitis, or strabismus, as well as those with a history of intraocular surgery or trauma, and those with a family history of glaucoma, were excluded from the study.

Demographic data, including age, gender, disease duration, medication use, and family history, as well as detailed clinical examination findings, were collected from the medical records of all participants. Additionally, parameters such as skin prick test results, serum total IgE levels, eosinophil count, and C-reactive protein (CRP) levels were recorded. Detailed ocular examination findings, including best corrected visual acuity (BCVA) assessed with the Snellen chart, intraocular pressure (IOP) measured with a non-contact tonometer, central corneal thickness, and anterior and posterior segment examination results obtained through slit-lamp biomicroscopy, were also evaluated. Posterior segment neuronal and vascular parameters, measured by OCT and OCTA, were obtained from patient records. For each participant, only one randomly selected eye was included in the study.

### Skin‐prick test

Skin prick tests were performed in the Pediatric Allergy Clinic laboratory using standard allergen kits from ALK-Abello (Horsholm, Denmark) and disposable plastic lancets (Stallergenes, Antony, France). The allergens were applied to the volar surface of both forearms, spaced at least 2 cm apart, with different lancets used for each allergen to ensure the allergens penetrated to a depth of approximately 1 mm. Histamine 0.1% (1 mg/mL) was used as the positive control, while saline solution was used as the negative control. A result was considered positive for the respective allergen if edema of 3 mm or more was observed compared to the negative control, 15 min after allergen application. The skin prick test panel included Dermatophagoides farinae, Dermatophagoides pteronyssinus, and a variety of pollen allergens, including grass pollen mix (Dactylis, Festuca, Lolium, Phleum, Poa), cereal pollen mix (Avena, Hordeum, Triticum, Secale), weed pollen mix (Artemisia, Chenopodium, Parietaria, Plantago), tree pollen mix (Alnus, Betula, Corylus), as well as olive tree pollen and Plantago pollen. Additionally, Alternaria, cat allergen, and dog allergen were tested. Sensitivity to house dust mites was defined as any reactivity to Dermatophagoides species, while sensitivity to grass, cereal, tree, and weed pollen was determined by any positive reaction to their respective pollen mixes.

### Total IgE and eosinophil counts

Total serum IgE levels were determined using an immunoenzymometric sequential assay ELISA kit; values exceeding 100 IU/mL were considered elevated. The total eosinophil count and the percentage of eosinophils in the blood were measured using an automated blood analyzer, the ABX Pentra 80 (Horiba Medical, Montpellier, France), and values greater than 4% were classified as elevated.

### Optic coherence tomography and optic coherence tomography angiography

OCT and OCTA images (DRI OCT Triton, Topcon, Japan) of all participants were analyzed. The OCT images were evaluated in two areas: the fovea, defined as a 1 mm × 1 mm square, and the parafoveal ring, defined as a 3 mm × 3 mm area. Central foveal thickness (CFT) and the thicknesses of the four parafoveal quadrants (nasal, superior, temporal, and inferior) and macular thickness (MT) were automatically measured by the device. A circle with a diameter of 3.45 mm was placed at the center of the optic disc, and a circular ring of 1.0 mm width around the optic disc boundary was considered the peripapillary region. The average peripapillary retinal nerve fiber layer (RNFL) thickness and the average thicknesses of the four peripapillary quadrants were determined using an automatic segmentation method. Images with a signal strength of ± 7 or less were excluded from the analysis. The subfoveal choroidal thickness (SCT) was manually assessed on the horizontal B-scan obtained via OCT, extending from the fovea to the inner surface of the sclera along the Bruch’s membrane.

**Fig. 1:**
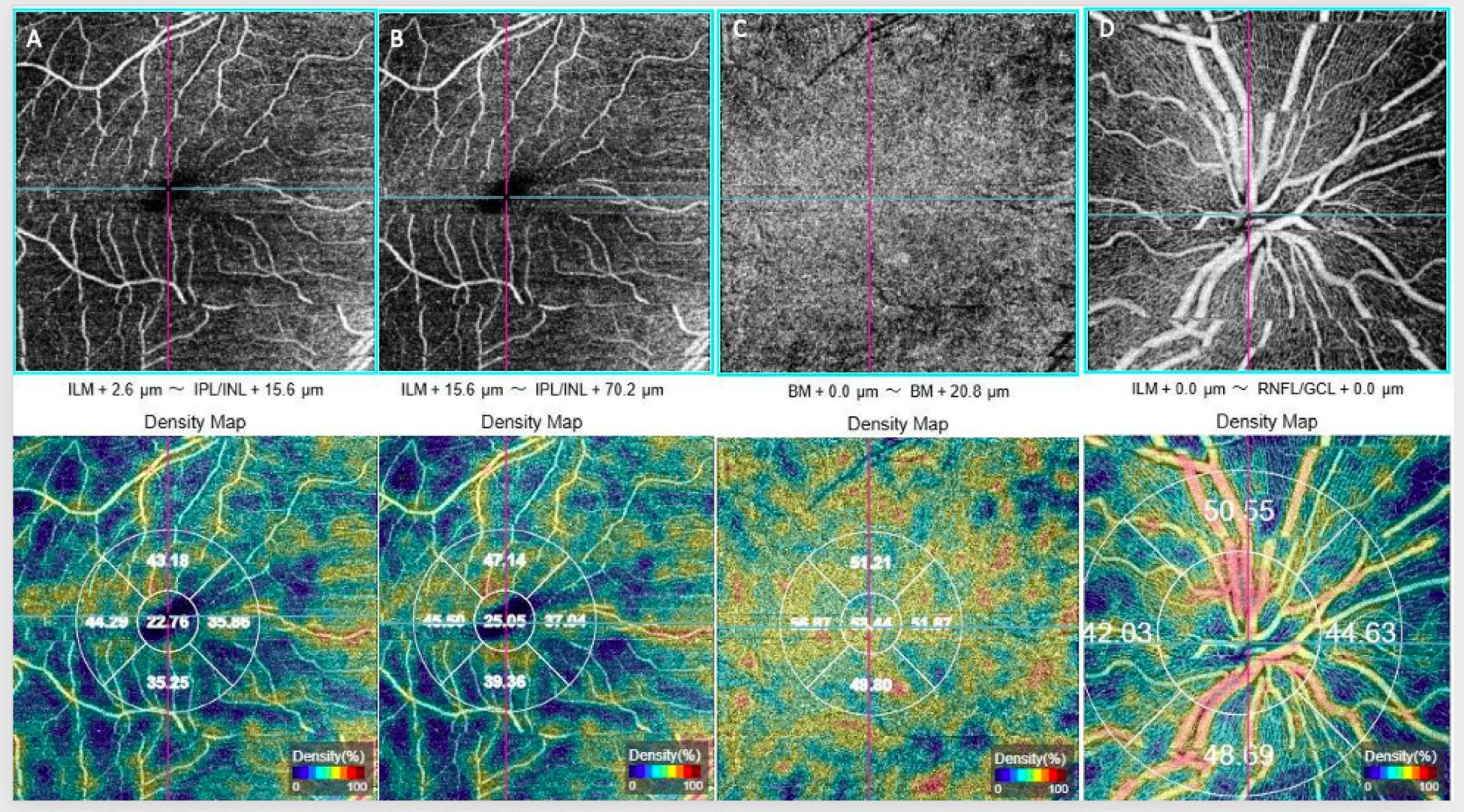
(**A**) Superficial vascular plexus, (**B**) deep vascular plexus, (**C**) choriocapillary vascular plexus, (**D**) peripapillary vascular plexus.

En-face images of the central macula area, measuring 6 × 6 mm^2^, were obtained using OCTA (DRI OCT Triton Plus, Topcon, Japan). These images were analyzed to measure the superficial capillary plexus (SCP), deep capillary plexus (DCP), and choriocapillary vascular plexus using Topcon’s IMAGEnet software. Vascular density (VD) was defined as the percentage of the area occupied by vessels. Two independent researchers manually delineated the area of the foveal avascular zone (FAZ), and the average of the measurements was recorded. Additionally, peripapillary vessel density (PVD) was assessed using a 4.5 × 4.5 mm^2^ rectangular scan centered on the optic nerve head, focusing on both the superficial and deep retinal layers. The SCP extends from 2.6 µm below the internal limiting membrane (ILM) to 15.6 µm below the inner plexiform layer (IPL), while the DCP spans from 15.6 µm below the IPL to 70.2 µm below the IPL. The outer retinal network extends from 70.2 µm below the IPL to Bruch’s membrane, while the choriocapillary network continues from Bruch’s membrane to 20.2 µm below it (Fig. 1). Images included in the study were selected after excluding those with a signal strength index (SSI) < 45, segmentation errors, or motion artifacts.

### Statistics

The data were presented as mean ± standard deviation, median (interquartile range), and n (%). Normality and homogeneity of variance were assessed using the Kolmogorov–Smirnov and Levene’s tests. To compare three groups with normally distributed data and homogeneous or non-homogeneous variances, Fisher’s ANOVA and Welch’s ANOVA tests were used, respectively. For non-normally distributed data, the Kruskal–Wallis test was applied. All statistical analyses were performed using the JAMOVI 2.3.18 software, and a *p* value < 0.05 was considered statistically significant.

## Results

A total of 39 asthma patients not using inhaled corticosteroids (Group 1), 47 asthma patients receiving inhaled corticosteroid treatment (Group 2), and 45 healthy controls were included in the study. The mean age was 11.67 ± 4.35 years for Group 1, 10.95 ± 3.45 years for Group 2, and 11.737 ± 4.68 years for the control group (*p* = 0.645). There was no significant difference in terms of age and gender between the participants. There were no statistical differences between Group 1 and Group 2 in terms of eosinophil count, eosinophil percentage, white blood cell count (WBC), total IgE, and CRP levels. The mean duration of inhaled corticosteroid use in Group 2 was 2.34 ± 1.75 years. Demographic and clinical characteristics of the participants are presented in Table 1.

**Table 1:** Clinical and demographic characteristics of the groups.

	Group1, no med (n = 39)	Group 2, steroid (n = 47)	Healthy controls (n = 45)	*p*
Age (years)	11.67 ± 4.35	10.95 ± 3.45	11.737 ± 4.68	0.645
Gender (Female)	21 (53.8%)	23 (48.9%)	27 (60%)	0.567
Refraction error, SE, D	− 0.25 (− 1.0 to 0.38)	− 0.50 (− 0.75 to 0.38)	− 0.75 (− 2.5 to 0.0)	0.033
Family history of atopy (n, %)	18 (46.2%)	25 (53.2%)	–	0.670
Disease duration (years)	4.0 (2.0–6.0)	4.0 (3.0–6.5)	–	0.265
IOP (mmHg)	16.0 (15.0–18.5)	17.0 (16.0–18.0)	17.0 (15.0–19.0)	0.624
Pachymetry (mm)	565.13 ± 25.09	571.95 ± 31.43	562.74 ± 30.16	0.293
Eosinophil counts	240 (125–530)	260 (110–470)	–	0.828
Eosinophil percentage	3.0 (1.8–7.1)	3.4 (1.6–6.6)	–	0.986
IgE	201 (39–304)	155 (84–421)	–	0.535
CRP	0.6 (0–1.4)	0.4 (0–1.9)	–	0.615
WBC	7.8 (7.1–8.7)	7.4 (6.7–8.7)	–	0.259
The time of drug use (years)	–	2.0 (1.0–2.5)	–	–

*CRP* C-reaktive protein, *D* diopter, *IOP* intraocular pressure, *IgE* immunglobulin E, *SE* spherical equivalent, *WBC* white blood count.

There were no statistically significant differences between the groups regarding the average CFT values and the mean values of RNFL and MT across the four quadrants (superior, inferior, temporal, and nasal) (*p* > 0.05 for all). However, there was a statistically significant difference between the groups in SCT (*p* < 0.001). SCT was significantly lower in Groups 1 and 2 compared to the control group, with Group 2 showing a thinner SCT. No statistically significant difference was found between Group 1 and Group 2 (*p* = 0.045 for Group 1 vs. control; *p* < 0.001 for Group 2 vs. control; *p* = 0.160 for Group 1 vs. Group 2) (Table 2).

**Table 2:** Comparison of peripapillary retinal nerve fiber layer thickness, macular thickness and subfoveal choroidal thickness values between the groups.

	Group1, no med (n = 39)	Group 2, steroid (n = 47)	Healthy controls (n = 45)	*p*
Average RNFL (µm) mean ± SD	106.72 ± 7.65	104.47 ± 9.65	103.46 ± 6.78	0.164
Superior quadrant RNFL (µm) mean ± SD	132.82 ± 11.95	129.67 ± 10.51	132.27 ± 14.36	0.441
Inferior quadrant RNFL (µm) mean ± SD	135.24 ± 17.93	129.58 ± 18.33	135.54 ± 13.57	0.165
Temporal quadrant RNFL (µm) mean ± SD	76.73 ± 9.94	77.35 ± 8.28	80.06 ± 11.93	0.257
Nasal quadrant RNFL (µm) mean ± SD	82.23 ± 11.45	80.53 ± 15.85	84.03 ± 12.58	0.475
Central MT (µm)	242 (222–267)	246 (232–257)	235 (226–247)	0.419
Superior quadrant MT (µm)	308.63 ± 18.05	304.05 ± 26.54	309.52 ± 13.82	0.391
Inferior quadrant MT (µm)	302.23 ± 22.84	300.95 ± 19.40	304.54 ± 13.56	0.573
Temporal quadrant MT (µm)	297 (287–308)	299 (284–309)	295 (286–305)	0.918
Nasal quadrant MT (µm)	307.15 ± 19.90	301.14 ± 20.62	309.38 ± 20.05	0.129
SCT (µm)	309.02 ± 61.19	286.62 ± 48.58	349.18 ± 88.86	**< 0.001**

Significant values are in [bold].

*MT* macular thickness, *RNFL* retinal nerve fiber layer, *SCT* subfoveal choroidal thickness, *SD* standart deviation.

VD analysis using OCTA revealed significant differences between the groups in the superior and nasal quadrants of the SCP (*p* = 0.007 and *p* = 0.005, respectively). Specifically, a significant difference was observed between Group 1 and the control group in the superior quadrant (*p* = 0.006), while in the nasal quadrant, a significant difference was found between Group 2 and the control group (*p* = 0.005). In contrast, the DCP showed statistically significant differences across all quadrants among the three groups (*p* < 0.001 for all). Notably, significant differences in the DCP were detected between Group 1 and the control group, as well as between Group 2 and the control group, across all quadrants (*p* < 0.001 for all). No statistically significant differences were identified among the three groups in terms of the superficial and deep FAZ area parameters (*p* = 0.206 and *p* = 0.766, respectively). Furthermore, significant differences were identified in the VD of the foveal choriocapillaris among the three groups, particularly between Group 1 and the control group (*p* = 0.043, *p* = 0.038, respectively). In the inferior and nasal quadrants of PVD, a significant differences were found among the three groups (*p* < 0.001), specifically between Group 1 and control group; as well as between Group 2 and the control group in the inferior quadrant (*p* < 0.002, *p* = 0.001, respectively), and between both groups and the control group in the nasal quadrant (*p* < 0.001 for all) (Table 3).

**Table 3:** Comparison of superficial and deep macular, peripapillary, and choricapillary vascular plexus of the groups and comparison of foveal avascular zone area among the groups.

	Group1, no med (n = 39)	Group 2, steroid (n = 47)	Healthy controls (n = 45)	*p*
SCP VD (%)				
Foveal	19.6 (17.3–21.6)	20.5 (18.1–23.9)	20.2 (18.3–23.5)	0.520
Superior	43.1 (40.5–45.2)	44.2 (40.1–45.8)	45.6 (42.9–47.5)	**0.007**
Inferior	42.13 ± 4.12	40.55 ± 4.98	42.15 ± 4.67	0.143
Temporal	43.80 ± 3.52	43.39 ± 3.55	44.83 ± 3.82	0.132
Nasal	41.57 ± 3.24	40.83 ± 4.62	43.65 ± 4.58	**0.005**
DCP VD (%)				
Foveal	19.1 (15.2–21.6)	19.1(16.1–24.1)	26.3 (22.4–29.1)	** < 0.001**
Superior	46.7 (44.1–49.6)	48.0 (44.0–51.4)	43.4 (38.6–44.9)	** < 0.001**
Inferior	42.07 ± 5.98	39.43 ± 4.78	45.15 ± 5.16	**< 0.001**
Temporal	41.42 ± 6.24	41.28 ± 4.90	46.39 ± 4.85	**< 0.001**
Nasal	45.3 (42.6–47.8)	45.2 (41.2–47.8)	42.2 (40.1–43.5)	**< 0.001**
FAZ (µm^2^)				
Superficial	339.20 ± 113.84	308.29 ± 106.29	345.16 ± 100.55	0.206
Deep	358.43 ± 124.82	347.50 ± 127.36	364.65 ± 83.57	0.766
Choriocapillaris VD (%)				
Foveal	52.3 (50.9–54.0)	53.5 (49.7–55.0)	54.1 (52.9–55.9)	**0.043**
Superior	52.05 ± 2.46	51.56 ± 3.33	51.78 ± 2.85	0.736
Inferior	52.25 ± 3.81	52.29 ± 3.83	52.26 ± 3.65	0.989
Temporal	52.12 ± 2.60	53.80 ± 2.64	54.23 ± 2.36	0.557
Nasal	52.53 ± 2.53	53.18 ± 3.15	53.94 ± 2.01	0.062
PVD (%)				
Superior	49.8 (49.6–51.0)	49.8 (49.6–50.3)	49.9 (49.8–50.0)	0.264
Inferior	51.0 (50.8–53.0)	50.9 (50.7–52.1)	50.3 (50.2–50.3)	**< 0.001**
Temporal	46.1 (45.3–47.6)	46.1 (45.2 (46.5)	45.7 (45.6–45.8)	0.075
Nasal	43.6 (43.0–44.0)	43.8 (43.1–44.5)	45.9 (45.8–45.9)	**< 0.001**

Median and Mean ± standard deviation values are shown.

Significant values are in [bold].

*DCP* deep capillary plexus, *FAZ* foveal avascular zone, *PVD* peripapillary vascular density, *SCP* superficial capillary plexus, *VD* vascular density.

A statistically significant negative correlation was observed between disease duration and mean RNFL, as well as between a family history of asthma and temporal quadrant RNFL (rho = − 0.215, *p* < 0.05; rho = − 0.303, *p* < 0.01, respectively). Additionally, a significant positive correlation was found between eosinophil count and PVD in the superior and temporal quadrants, as well as choriocapillaris VD in the inferior quadrant (rho = 0.154, *p* < 0.05; rho = 0.181, *p* < 0.05; rho = 0.179, *p* < 0.05, respectively). A significant negative correlation was also observed between CRP levels and foveal SCP and DCP (r = − 0.180, *p* < 0.05; r = − 0.215, *p* < 0.01, respectively).

## Discussion

Asthma is not solely a condition affecting the respiratory system; it also has implications for ocular health. The prevalence of ocular manifestations in asthma patients is significant and may be related not only to the underlying pathophysiology of the disease itself but also to the treatments employed^15,16^. Recognizing ocular changes, understanding their etiology, and ensuring both their prevention and management can significantly contribute to improving the quality of life in individuals with asthma. To the best of our knowledge, this study is the first to evaluate the effects of asthma comprehensively and inhaled corticosteroids on posterior segment parameters, including the macula, optic nerve, and choroidal parameters, by comparing pediatric asthma patients receiving and not receiving inhaled corticosteroid therapy with a healthy control group. In this study, a statistically significant reduction was observed in the SCT, as well as in the SCP in the superior and nasal quadrants, in the DCP in all quadrants, the choriocapillaris in the foveal quadrant, and the PVD in the inferior and superior quadrants in the asthma group.

Several studies in the literature have examined the RNFL, CFT, and SCT in pediatric patients with asthma^17–19^. Dereci et al. found that, in pediatric asthma patients treated with inhaled corticosteroids for approximately 1.6 years, the RNFL thickness was similar to the control group, while CFT was significantly thicker. They also observed a significant negative correlation between CFT and disease duration^17^. Günay et al. reported that in asthmatic children using inhaled corticosteroids, the superior, inferior, and average RNFL thicknesses were thinner compared to the control group, while no significant difference was found in CFT and SCT. Additionally, they identified a significant negative correlation between RNFL thickness and corticosteroid dosage^18^. Karaaslan et al. observed a significant thinning of the RNFL in asthmatic children compared to the control group, particularly in those not using corticosteroids. However, no significant difference in CFT was found between asthmatic patients using or not using corticosteroids and the control group. They suggested that the thinning of the RNFL might be more closely related to chronic hypoxia resulting from the disease, as well as systemic inflammation, cytokine release, and alterations in the oxidant-antioxidant balance activated by the disease, rather than the use of inhaled corticosteroids^19^. In our study, no significant differences were found in RNFL and CFT between the pediatric asthma group (both steroid users and non-users) and the healthy control group. However, SCT was found to be significantly thinner in the asthma group. Although both steroid-treated and untreated asthma patients differed significantly from the control group, no statistically significant difference was found between the steroid-treated and untreated asthma groups. In our study, it was considered that the alterations in SCT were more attributable to the underlying disease than to the use of inhaled corticosteroids. Kurultay et al. observed that during the attack phase, the thickness of the SCT increased; however, after treatment, it returned to levels similar to those of the control group^20^. Yılmaz et al. found that in a stable adult asthma group who had received corticosteroid treatment for the past 2 months, no statistically significant difference in RNFL was observed compared to the control group^21^. However, they reported a significant reduction in both SCT and the choroidal vascular index. This has been linked to increased levels of cytokines, such as IL-18, IL-6, TNF-α, leukotrienes, and chemokines, in patients with asthma. These cytokines interact with cells like platelets, endothelial cells, erythrocytes, and leukocytes, contributing to the formation of cell aggregates and atherosclerotic plaques. Additionally, it has been suggested that the increased sympathetic system overactivation in asthma patients leads to vasoconstriction, further contributing to this process^21^. Aligned with the results of this study, we hypothesized that the vascular structures could be affected due to hypoxia, hypercapnia, systemic inflammatory processes, increased oxidative stress, and sympathetic system activation associated with asthma. Consequently, we suggested that choroidal circulation might be impaired, leading to a reduction in choroidal thickness. We attributed the varying results in RNFL and CFT across studies to differences in disease duration, severity, and variations in the inflammatory responses related to the disease among patients. In our study, we identified a statistically significant negative correlation only between disease duration and average RNFL, as well as between a family history of asthma and the temporal quadrant of the RNFL. Although we did not observe a significant change in RNFL compared to the control group, we considered that, in the long term, inflammatory and hypoxic processes associated with the disease might lead to alterations in RNFL. Furthermore, we hypothesized that individuals with a family history of asthma may exhibit stronger immunological and vascular responses compared to those without such a history. The use of inhaled corticosteroids may also contribute to this process over time.

It is well established that retinal microvascular structures are affected by diseases associated with inflammation and hypoxic processes^22–24^. Yi et al. demonstrated a statistically significant decrease in VD in both the SCP and DCP during both active and quiescent phases in pediatric uveitic patients. They attributed these findings to damage in the inner and outer retinal barriers secondary to subclinical inflammation in the posterior segment. Additionally, Yang et al. found that in asthma mouse models, inflammation associated with both acute and chronic processes triggered the TGF-β1/Smad signaling pathway, leading to the development of choroidal neovascular membranes, with this effect being particularly pronounced in the chronic asthma group^25^. Ye et al. observed a significant increase in VD in both SCP and DCP following adenotonsillectomy in children with obstructive sleep apnea syndrome (OSAS) due to adenoid hypertrophy^26^. They also noted a significant reduction in the FAZ width but no significant change in peripapillary VD. They suggested that the hypoxia, hypercapnia, and acidosis associated with OSAS could lead to damage, especially in macular VD, which is more susceptible to hypoxia. Yu et al. reported that in adult patients with OSAS, peripapillary and parafoveal VD decreased in accordance with disease severity^24^. Karaaslan et al. in their OCTA study, evaluated macular and peripapillary VD parameters in pediatric asthma patients compared to a control group^19^. They observed a reduction in flow area in the outer retina of the macula and an increase in inside disc density. In our study, which assessed macular, peripapillary, and choroidal VD using OCTA in both corticosteroid-treated and untreated asthma patients compared to a control group, we found that VD in the superior and nasal quadrants of the SCP and all quadrants of DCP, as well as in the choriocapillaris in the foveal region, and the inferior and nasal peripapillary VD, were significantly reduced in the asthma group compared to the control group. The lack of significant differences in vascular structures between the inhaled corticosteroid-treated and untreated groups was attributed to the fact that changes in retinal microvascular structures may be more closely related to the disease itself rather than steroid use. Moreover, the negative correlations observed between eosinophil count and vessel density (VD), as well as between C-reactive protein (CRP) and VD, further support this hypothesis. Elevated eosinophil counts and CRP levels play a critical role in the frequency and severity of asthma exacerbations and overall asthma prognosis^27,28^. We found that changes in inflammatory and hypoxic processes associated with asthma pathogenesis may lead to a reduction in retinal vascular tissues correlated with the severity of asthma. Consequently, there may be an increased risk of developing certain retinal, optic nerve, and choroidal diseases during long-term follow-up in these children.

The retrospective design of this study, the small sample size, and the relatively short follow-up period represent the limitations of the study. We believe that future prospective studies with a larger sample size and longer follow-up duration would be valuable in evaluating the posterior segment changes associated with asthma.

In conclusion, the pathophysiological mechanisms of asthma, such as hypoxia, systemic inflammation, and pharmacological treatments, may lead to reduced vascular density in the microvascular structures of the posterior segment of the eye, particularly in the SCP, DCP, PVD, and choriocapillary VD, as well as a decrease in SCT. These changes did not show statistically significant differences between the groups receiving inhaled steroid treatment and those not receiving it. These alterations have the potential to cause significant long-term impairments in visual function among pediatric patients with asthma. Considering these possible consequences, regular monitoring of children with asthma using non-invasive, efficient, and reliable imaging modalities, such as OCT and OCTA, is crucial.

## Data Availability

The datasets generated and/or analysed during the current study are not publicly available due [Due to the retrospective nature of this study and the absence of informed consent from the patients, the data is not publicly available for ethical reasons.] but are available from the corresponding author on reasonable request.
